# Genome-Wide Association Study Reveals Genetic Mechanisms Underlying Intersex and Aproctia in Large White Pigs

**DOI:** 10.3390/ani15081094

**Published:** 2025-04-10

**Authors:** Yajun Li, Jiaxin Shi, Yingshan Yang, Donglin Ruan, Jie Wu, Danyang Lin, Zihao Liao, Xinrun Hong, Fuchen Zhou, Langqing Liu, Jie Yang, Ming Yang, Enqin Zheng, Zhenfang Wu, Gengyuan Cai, Zebin Zhang

**Affiliations:** 1 State Key Laboratory of Swine and Poultry Breeding Industry, National Engineering Research Center for Breeding Swine Industry, State Regional Livestock and Poultry Genebank, Guangdong Genebank of Livestock and Poultry, College of Animal Science, South China Agricultural University, Guangzhou 510642, China; liyajun202207@163.com (Y.L.); shijiaxinkuna@163.com (J.S.); y768238376@163.com (Y.Y.); ruandl@stu.scau.edu.cn (D.R.); wujiezi163@163.com.com (J.W.); danyanglinldy@163.com (D.L.); gou9857143ji@163.com (Z.L.); h596676148h@163.com (X.H.); zfc17854225519@163.com (F.Z.); langqing.liu@scau.edu.cn (L.L.); jieyang2012@hotmail.com (J.Y.); eqzheng@scau.edu.cn (E.Z.); wzfemail@163.com (Z.W.); 2 Guangdong Provincial Key Laboratory of Agro-Animal Genomics and Molecular Breeding, South China Agricultural University, Guangzhou 510642, China; 3 Guangdong Key Laboratory of Animal Breeding and Nutrition, Institute of Animal Science, Guangdong Academy of Agricultural Sciences, Guangzhou 510640, China; 4 College of Animal Science and Technology, Zhongkai University of Agriculture and Engineering, Guangzhou 510225, China; yangming@zhku.edu.cn; 5 Yunfu Subcenter of Guangdong Laboratory for Lingnan Modern Agriculture, Yunfu 527300, China; 6 Guangdong Zhongxin Breeding Technology Co., Ltd., Guangzhou 510642, China

**Keywords:** intersex, aproctia, chip data, imputed data, mixed linear models, genome-wide association studies

## Abstract

Congenital developmental abnormalities in piglets, such as intersex and aproctia, adversely affect survival rates, growth performance, and genetic breeding efficiency in pig populations. To elucidate their genetic basis, we performed a genome-wide association study (GWAS) on 1030 Large White pigs. We combined 50 K SNP chip data with SWIM-based genotype imputation to enhance the resolution of genetic variation detection, followed by MLM analysis. Our results identified 53 significant SNPs, with 52 associated with intersex and 1 with aproctia. Key candidate genes included MAD1L1, ID4, EFNA5, and PPP1R16B for intersex and ARNT2 for aproctia. Functional enrichment analysis highlighted pathways related to gonadal development (e.g., progesterone-mediated oocyte maturation) and embryonic morphogenesis. Collectively, the identification of these SNPs and candidate genes advances our understanding of the genetic architecture of intersex and aproctia in piglets. These findings provide actionable insights for optimizing genetic breeding strategies and improving health management in Large White pig production, with potential implications for reducing economic losses caused by congenital disorders.

## Simple Summary

We conducted a genome-wide association study (GWAS) on 1030 Large White pigs to investigate the genetic mechanisms underlying congenital developmental abnormalities, specifically intersex and aproctia. We applied mixed linear models (MLMs) to identify genetic variants significantly associated with these traits using both chip data and SWIM-imputed data. A total of 53 significant single-nucleotide polymorphisms (SNPs) were identified, with 52 linked to intersex and 1 to aproctia. Functional annotation revealed fifteen candidate genes for intersex (e.g., *MAD1L1*, *ID4*, and *EFNA5*) and four for aproctia (e.g., ARNT2). These findings enhance our understanding of the genetic mechanisms underlying intersex and aproctia in piglets and provide novel insights for further exploration of the genetic basis of these congenital developmental disorders. The identified markers may serve as potential targets for molecular breeding programs aimed at reducing piglet mortality and improving production efficiency.

## 1. Introduction

In pig genetic breeding programs, intersex and aproctia are critical congenital anomalies that adversely affect population health and productivity. Disorders of sex development (DSD), a group of congenital conditions characterized by chromosomal, gonadal, or anatomical sex abnormalities [1], cause the abnormal development of reproductive organs, thus affecting the reproductive capacity of individuals. Intersex pigs, which exhibit a female chromosomal karyotype (38,XX) but lack the sex-determining region Y (SRY) gene, are classified as 38,XX-DSD (SRY-negative) and represent a type of DSD. These disruptions impair reproductive organ development and individual fertility [2]. Affected individuals often display ambiguous genitalia, including a hypertrophied clitoris, vulvar malformations, and occasionally scrotal-like structures or penile enlargement, despite a predominantly female chromosomal profile [3]. The offspring of carrier boars exhibit intersex incidence rates exceeding 4–5% [3], resulting in infertility, behavioral aggression during fattening, and economic losses due to compromised carcass quality [4]. Although intersexuality has been well studied in horses [5] and cattle [6], the genetic mechanisms underlying porcine DSD remain poorly characterized, with only a few candidate genes identified.

Congenital aproctia, a rare gastrointestinal malformation affecting approximately 0.02–0.06% of the global population, exhibits a male-biased sex ratio and familial aggregation in 1–9% of cases. Although environmental influences cannot be ruled out, genetic factors are the primary determinants of aproctia susceptibility, as evidenced by heritability estimates ranging from 0.11 to 0.35 in pig populations [7,8]. Genome-wide studies in Pietrain × Landrace crosses have mapped aproctia-associated loci to sus scrofa chromosome1 (SSC1), SSC9, and SSC12, involving genes such as *FMN*, *BMP4*, *HOXB5*, and *HOXB9*, suggesting that the disease may be controlled by single-gene or multi-gene inheritance [9]. In addition, other studies have also identified candidate pathogenic genes such as *GLI2* [10,11] and *IHH* [11] on SSC15 of Landrace pigs and Large White pigs. In addition, a survey of congenital malformations in Australia found that the overall incidence of the disease was 0.27% [12]. This disease is particularly serious in boars because it usually causes death, usually within 1 to 3 days. Although surgical correction is the only treatment, the postoperative survival rate is low. For sick sows, some individuals will form rectovaginal fistulas, meaning that feces can be discharged from the vaginal opening, so some sows can survive, and their survival rate within 1 month of age is about 50% [13]. A congenital imperforate anus has adverse effects on pig health, breeding efficiency, and animal welfare, including acute intestinal obstruction, secondary infection, increased mortality, and decreased reproductive efficiency.

Despite the economic and welfare implications of intersex and aproctia in pig production, the genetic architecture of these anomalies remains incompletely characterized, hindering the development of molecular screening tools. This study aims to address this gap by conducting a genome-wide association study (GWAS) in a large cohort of 1030 Large White pigs, integrating high-density SNP data and imputation strategies to identify novel candidate genes and pathways underlying these developmental disorders. In addition, since pigs have certain similarities with humans in physiological and genetic characteristics, the results of this study can also provide valuable references for the study of related human diseases.

## 2. Materials and Methods

### 2.1. Ethics Statement

All animals used in this study were treated in accordance with the guidelines for the use of laboratory animals of the Ministry of Agriculture of China and with the approval of South China Agricultural University (Guangzhou, China), No. 2018F089.

### 2.2. Animals and Phenotypic Collection

The data used in this study were all from existing laboratory resources, and no additional collection was required. The genotype and phenotype data of Large White pigs were collected from 12 different farms under Guangdong Wens Food Co., Ltd. We collected a total of 1030 piglets, 21 of which were affected by intersex conditions and 70 had aproctia. The remaining 939 normal piglets were used as a case–control in the subsequent analyses of intersex and aproctia. All sows were raised and farrowed under the same environmental conditions. A unified feeding and management model was adopted throughout the fattening stage. All pigs received the same dietary formula and had free access to water. The temperature was controlled between 18 °C and 22 °C to minimize the impact of environmental factors on trait determination. All diseased piglets were identified by on-site professionals. Intersex individuals were identified based on abnormalities in the external genitalia and reproductive tract structure, while aproctia was identified based on the presence of anal atresia after birth.

### 2.3. Genotype Data Acquisition and Quality Control

Genomic DNA was extracted from the tail tissues of 1030 piglets using the phenol-chloroform method. Following extraction, DNA quality was assessed through three criteria: (1) spectrophotometric evaluation of light absorption ratios (A260/280 and A260/230), (2) gel electrophoresis analysis, and (3) DNA concentration quantification (50 ng/μL) to meet experimental standards. Genotyping was performed using the GenoBaits Porcine 50 K SNP Panel. The generated VCF files were converted to binary PED format using PLINK (v1.90) [14]. Variants with >10% missingness or minor allele frequency (MAF) < 1% were excluded. Individuals were excluded if they met any of the following criteria: (1) >10% missing genotypes, (2) relatedness (kinship coefficient > 0.125), (3) sex mismatch, or (4) outliers identified through PCA based on 960 and 1009 reference genomes, respectively [15]. All variants were subjected to a Hardy–Weinberg equilibrium (HWE) exact test, and those deviating from HWE (*p* < 1 × 10^−5^) were removed. After stringent quality control, 960 intersex individuals (939 controls, 21 cases) and 1009 aproctia individuals (939 controls, 70 cases) were retained, yielding 47,685 and 45,576 high-quality SNPs for subsequent analyses, respectively.

To increase marker density, genotype imputation was performed using the SWIM server (https://quantgenet.msu.edu/swim/, accessed on 14 December 2023) [16] with default parameters. The reference haplotype panel comprised whole-genome sequencing data from 2259 pigs spanning 44 breeds. Imputation accuracy was evaluated by three metrics: (1) average consistency rate (97%), (2) non-reference consistency rate (91%), and (3) squared correlation coefficient (r^2^ = 0.89), collectively confirming high data reliability. Following imputation and quality control, 30,489,782 high-confidence SNPs were retained for subsequent association analyses of both intersex and aproctia traits.

### 2.4. Population Genetics and Fst Analysis

To investigate the genetic structure of the study population, we performed PCA on 960 intersex individuals and 1009 individuals with aproctia using GCTA v1.93 [17]. To further assess the genetic differentiation between the case and control groups, we estimated the Fst value using the Weir and Cockerham method implemented in VCFtools. For the 50 K chip data, Fst was estimated at each SNP variant site, whereas for the imputed data, it was computed across genomic regions using a sliding window of 50,000 bp with a 25,000 bp overlap [18].

### 2.5. Genome-Wide Association Analysis

To test the genetic effects of each variant on the intersex and aproctia traits, we performed GWAS. GWAS was performed using the GEMMA linear association model (v0.98) [19], running GEMMA on the joint dataset of all phenotypes. GWAS adjusted for sex and the first five genetic principal components. Then, the phenotypes and genotypes were input into the fixed-effect model (GEMMA v0.98) for GWAS analysis.

GEMMA is an effective tool for genome-wide complex trait analysis, capable of estimating variance components explained by genome-wide SNP markers and the heritability of body shape traits. GEMMA uses the restricted maximum-likelihood method to estimate the variance components. The model for estimating the variance components explained by genome-wide SNP markers was the following:

*y* = W*α* + X*β* + Z*μ* + *ε*

In the formula, *y* is the phenotype value; X is genotype; W is the association matrix of covariates, including the fixed effect of sex and the first five principal components of PCA analysis; *α* is a vector of the corresponding coefficients, including the intercept; Z is the genetic relationship matrix (GRM) between individuals calculated by GCTA; *β* is the fixed effects; *μ* is the random genetic effect of individuals, indicating the relatedness between individuals; and *ε* is the residual error.

### 2.6. Conditional Analysis

In genetic association analysis, the phenomenon of significant clustering of multiple SNPs in genomic association regions may be attributed to linkage disequilibrium (LD) between these loci. To investigate whether the distinct signal peaks within these regions represent independent associations, we performed conditional analysis using an MLM approach implemented in the GEMMA software (version 0.98.5). This involved iteratively incorporating the genotype of the lead SNP (defined as the variant with the most significant *p*-value) as a covariate in the model. Subsequent association signals were systematically evaluated through stepwise regression: after conditioning on the primary signal, residual associations were re-examined, and the process was repeated by sequentially conditioning on each newly identified lead SNP until no genome-wide significant signals remained. This approach allowed for the differentiation of independent association signals from those driven by LD with the primary variant.

### 2.7. Identification of Candidate Genes and Functional Enrichment Analysis

In this study, 50 K chip genotype data and SWIM imputed data were used to define a 500 kb genomic interval centered on each lead SNP as a candidate gene region. In order to explore the functions of the above genes, we used the latest version of the Sus scrofa 11.1 genome (http://ensembl.org/Sus_scrofa/Info/Index, accessed on 10 September 2023) as the reference genome and downloaded the functional gene annotation file (GFF3 format) of version (v105) from the Ensembl website (http://ftp.ensembl.org/pub/release-105/gff3/sus_scrofa/, accessed on 10 September 2023). The genes were then subjected to gene ontology (GO) terminology annotation, National Human Genome Research Institute (NHGRI) GWAS catalog annotation, and Kyoto Encyclopedia of Genes and Genomes (KEGG) [20] pathway analysis using KOBAS v3.0 [21]. We selected the enriched items with corrected *p*-values less than 0.05, used Fisher’s exact test and Benjamini–Hochberg correction [22], and further explored the biological pathways and processes these genes were involved in. At the same time, we manually searched for genes near significant loci in the literature and databases such as MGI (MGI-Mouse Genome Informatics-The international database resource for the laboratory mouse), GeneCards (GeneCards—Human Genes | Gene Database | Gene Search), and other relevant ones.

## 3. Results

### 3.1. Phenotyping and SNP Genotyping

**Figure 1:**
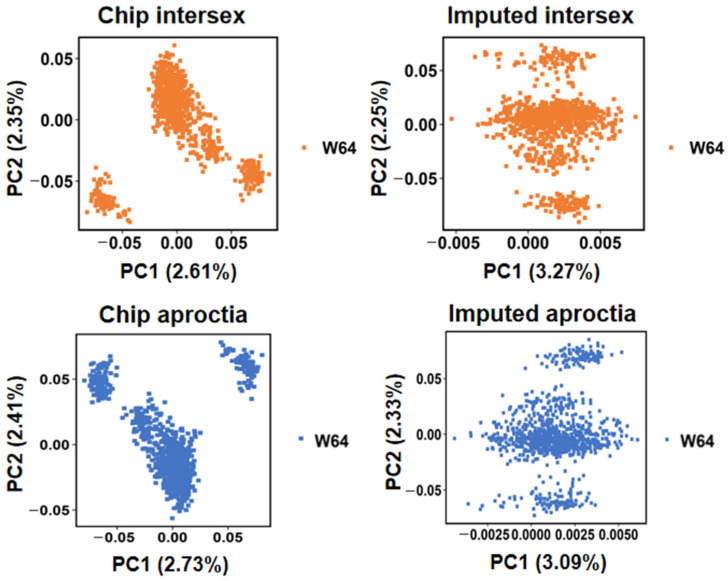
PCA plots of intersex and aproctia.

DNA was extracted from piglet tail tissue samples, and genotyping was performed using the GenoBaits Porcine 50 K SNP chip. Quality control of genotype data was performed using PLINK (v1.9). Following quality control procedures for both genotype and phenotype data, individuals with ambiguous phenotypic classifications were excluded. PCA was subsequently employed to account for population structure and remove genetic outliers (Figure 1). The phenotypic distributions of intersex and aproctia traits were statistically characterized separately. For the intersex trait, the dataset included 960 individuals, comprising 939 normal and 21 diseased individuals. For the aproctia trait, 1009 individuals were analyzed, consisting of 939 normal and 70 diseased individuals.

### 3.2. Genome-Wide Association Analysis Results

**Figure 2:**
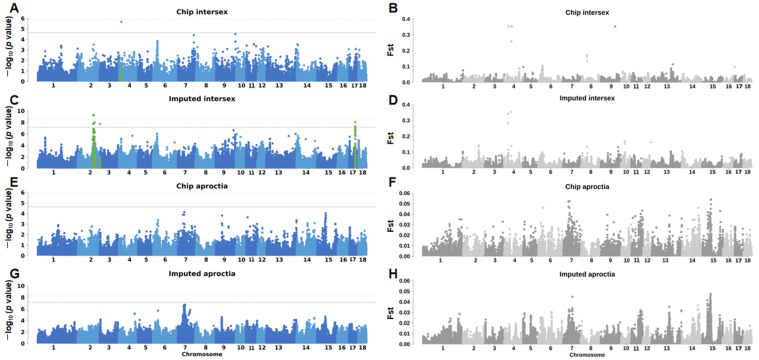
Manhattan plots and fst analysis results of intersex and aproctia: (**A**,**B**) intersex chip data; (**C**,**D**) intersex imputed data; (**E**,**F**) aproctia chip data; and (**G**,**H**) aproctia imputed data. The dotted line is the significance threshold of GWAS analysis (*p* = 1/n) (n is the number of effective SNPs, n_Chip intersex SNPs_ = 47,685, n_Chip aproctia SNPs_ = 45,576, and n_Imputed SNPs_ = 30,489,782). The green in the figure indicates the genetic variation site 500kb above and below the significant SNP.

In the GWAS analysis of chip data, we found only one significant association site in the intersex group (Figure 2A, Table S2), and no significant association sites were found in the aproctia group (Figure 2E, Table S4). In order to increase the genome coverage, enhance the statistical power, and discover potential functional variants, we performed GWAS analysis on the chip data imputed by SWIM. In the intersex imputed data, we observed more significant signal peaks and found more significant SNPs (Figure 2C, Table S1). Although the genetic variation in the aproctia did not reach the significant threshold at the genome-wide level, a clear signal peak was still formed on SSC 7 (Figure 2G, Table S3). Overall, in the intersex chip data, we found one significant SNP, and one gene was located within 500 kb of the SNP; in the imputed data analysis, 51 significant SNPs were found, and they were co-located to 14 genes within 500 kb. In the aproctia imputed data, one lead SNP was found, and four genes were co-located within 500 kb (Table 1).

**Figure 3:**
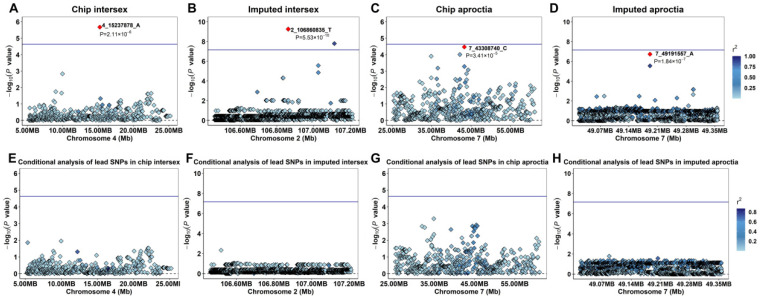
Manhattan plots and fst analysis results of intersex and aproctia: (**A**,**B**) intersex chip data; (**C**,**D**) intersex imputed data; (**E**,**F**) aproctia chip data; and (**G**,**H**) aproctia imputed data. The blue horizontal line is the significance threshold of analysis (*p* = 1/n) (n is the number of effective SNPs, n_Chip intersex SNPs_ = 47,685, n_Chip aproctia SNPs_ = 45,576, and n_Imputed SNPs_ = 30,489,782).

For further study, we used GEMMA software to fit the lead SNP (4_15237878_A) of the intersex chip data, the lead SNP (2_106860835_T) of the imputed data, the lead SNP (7_43308740_A) of the aproctia chip data, and the lead SNP (7_49191557_A) of the imputed data as covariates into the MLM and performed conditional analysis (Figure 3A–H). The results showed that the signal peaks in the adjacent range of the chromosomes where the lead SNPs of the two traits were located disappeared, and the *p*-values of the related SNPs all dropped below the threshold line, indicating that these SNPs have a strong linkage relationship with the lead SNP and are dominated by the lead SNP.

### 3.3. Fst Analysis Results

To quantify genetic differentiation between the case and control cohorts, we performed fixation index (Fst) analysis. This metric measures population divergence through allele frequency differences, ranging from 0 to 1. Given that GWAS are particularly vulnerable to population stratification effects which can generate spurious associations, we integrated Fst estimates to quantify population structure. These values informed subsequent GWAS model adjustments through PCA and environmental covariate inclusion. Notably, elevated Fst values suggest substantial genetic divergence potentially attributable to geographic barriers, selective pressures, or stochastic genetic drift processes. Loci exhibiting exceptional Fst elevations may represent selection targets, potentially influencing environmental adaptation or disease susceptibility. This complementary approach helps distinguish between GWAS signals arising from phenotypic association versus those reflecting neutral evolutionary processes.

In the chip data and imputed data of the aproctia trait, the most significant GWAS region and the most significant Fst locus were both located in the same region on SSC 7 (Figure 3F,H), which may indicate that the genetic variation in this region both affects the phenotype and is subject to selection pressure. In the intersexual trait, the most significant GWAS region and the most significant Fst locus in the chip data were both located on SSC 4 (Figure 3B,D); in the imputed data, although the most significant GWAS region and the most significant region in the Fst analysis were not in the same region, the Fst peak signal could be observed on SSC 2.

### 3.4. Functional Annotation of Candidate Genes

To further interpret the GWAS results, we annotated 15 genes located within 250 kb upstream and downstream of the SNPs identified in the intersex chip and imputed data. These included *ST8SIA4* (ST8 Alpha-N-Acetyl-Neuraminide Alpha-2,8-Sialyltransferase 4), *SLCO4C1* (Solute Carrier Organic Anion Transporter Family Member 4C1), *EFNA5* (Ephrin A5), *EPB41L4A* (Erythrocyte Membrane Protein Band 4.1 Like 4A), *MRM2* (Mitochondrial RRNA Methyltransferase 2), *SNX8* (Sorting Nexin 8), *EIF3B* (Eukaryotic Translation Initiation Factor 3 Subunit B), *ENSSSCG00000039023*, *CHST12* (Carbohydrate Sulfotransferase 12), *MAD1L1* (Mitotic Arrest Deficient 1 Like 1), *NUDT1* (Nudix Hydrolase 1), *FAM83D* (Family With Sequence Similarity 83 Member D), *PPP1R16B* (Protein Phosphatase 1 Regulatory Subunit 16B), *DHX35* (DEAH-Box Helicase 35), and *ID4* (Inhibitor Of DNA Binding 4). Pathway enrichment analysis revealed significant associations with intersex-related Kyoto Encyclopedia of Genes and Genomes (KEGG) and Gene Ontology (GO) terms, including progesterone-mediated oocyte maturation, TGF-beta signaling pathway, cellular response to follicle-stimulating hormone stimulus, prostate gland epithelium morphogenesis, and regulation of phosphatidylinositol 3-kinase signaling. These pathways are primarily implicated in sex differentiation, gonad development, and hormonal regulation.

**Figure 4:**
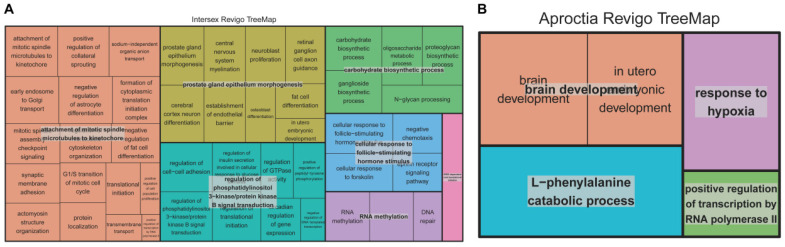
Non-redundancy GO terms for biological process category intersex and aproctia genes. (**A**) shows the “TreeMap” view of intersex REVIGO, while (**B**) shows the “TreeMap” view of aproctia REVIGO. Each rectangle represents a representative cluster. These representatives are grouped into “superclusters”, representing loosely related terms and visualized using different colors.

Non-redundant GO analysis via REVIGO further clustered key terms such as prostate gland epithelium morphogenesis, cellular response to follicle-stimulating hormone stimulus, and regulation of phosphatidylinositol 3-kinase/protein kinase B signal transduction (Figure 4A). These processes may collectively influence sex hormone dynamics and gonad development, potentially contributing to intersex phenotypes.

Similarly, four genes near the SNP linked to aproctia in the imputed data were annotated: *ZFAND6* (Zinc Finger AN1-Type Containing 6), *FAH* (Fumarylacetoacetate Hydrolase), *CTXND1* (Cortexin Domain Containing 1), and *ARNT2* (Aryl Hydrocarbon Receptor Nuclear Translocator 2). Pathway enrichment analysis highlighted terms such as “in utero embryonic development” aligning with the congenital nature of aproctia, which likely originates during early embryogenesis (Figure 4B).

In summary, these findings suggest that *MAD1L1*, *ID4*, *EFNA5*, and *PPP1R16B* are potential candidate genes for intersex, likely influencing sex hormone regulation and gonadal development, while *ARNT2* may contribute to aproctia through its role in embryonic development. Further functional studies are warranted to elucidate their precise roles.

## 4. Discussion

Intersex disorders in pig are pathologically characterized by reproductive system malformations, leading to infertility or subfertility which severely compromises porcine reproductive capacity. Congenital aproctia exacerbates neonatal mortality through fecal retention syndrome, directly diminishing litter viability and breeding efficiency. Together, these two congenital malformations impose significant constraints on commercial pork production systems. While both environmental and genetic factors contribute to their etiology, current evidence suggests genetic factors as predominant contributors [23,24,25,26,27]. Contemporary investigations have employed histological characterization and chromosomal karyotyping to delineate these anomalies [28,29,30,31,32], with emerging molecular studies identifying associated DNA signatures [33,34]. Nevertheless, the precise genetic determinants remain incompletely elucidated.

This study implemented GWAS to investigate intersex and aproctia in a Large White pig cohort (n = 1030), utilizing SNP chip data augmented by genotype imputation. To validate GWAS findings, we implemented Fst analysis. GWAS identified genes associated with target traits by statistically analyzing the association of SNPs, and FST analysis was used to assess whether these GWAS hotspots showed higher population genetic differentiation between the case and control groups. If the FST value was significantly increased near the GWAS significant SNP, it meant that the gene region had undergone significant genetic differentiation between the affected and unaffected groups, further supporting its association with the target trait. If the FST value of the GWAS signal region was low, it meant that the genetic variation in the population was small, which might have been due to the weak effect of the SNP or the association results in the GWAS might have been due to other factors (such as population structure). We found that significant SNPs had a trend of genetic differentiation. Finally, functional enrichment assessment was performed. Fourteen loci demonstrated significant associations with intersex phenotypes, whereas a single genomic region was associated with aproctia. Pathway analyses identified *MAD1L1*, *ID4*, *EFNA5*, and *PPP1R16B* as prime intersex candidates, with *ARNT2* emerging as a plausible aproctia-associated gene.

Hormone level is an important criterion for diagnosing DSD because it is often closely related to hormone secretion disorders, with intersex pigs exhibiting progesterone concentrations exceeding normal sow levels by 5–90 fold [35]. Testosterone and estradiol profiles in these individuals consistently occupy intermediate ranges between normal boars and sows. Notably, gonadal androstenone production in intersex subjects correlates with meat quality [36]. Ultrastructural analysis via transmission electron microscopy revealed pituitary hyperplasia in 5-month-old intersex pigs, characterized by the proliferation of electron-dense secretory granules in gonadotropin and prolactin cells, which indicated that there were abnormalities in the structure and function of the pituitary of intersex pigs. Therefore, when screening intersex candidate genes, we paid special attention to genes related to sex hormone secretion and gonadal development.

The mitotic checkpoint gene *MAD1L1* warrants particular attention, given its maternal polymorphisms’ correlation with fetal survival in chromosomal abnormalities [37] and association with advanced epithelial ovarian cancer [38]. Similarly, *ID4* maintains spermatogonial stem cell plasticity through the regulation of retinoic acid-mediated differentiation [39], while concurrently mediating estrogen/progesterone-responsive mammary ductal morphogenesis via *p38MAPK* inhibition [40,41]. Although these oncogenic associations may appear divergent, their shared regulatory roles in steroid hormone responsiveness provide plausible mechanisms for intersex pathogenesis.

Notably, while *ZFAND6*, *FAH*, and *CTXND1* lack established aproctia associations, *ARNT2* demonstrated role in embryogenesis [38] suggests potential involvement in cloacal formation, a developmental precursor to anal–rectal structures. This warrants targeted investigation given the embryonic origin of aproctia-related malformations. In actual pig breeding, if piglets with genetic defects are found, the staff usually eliminate the entire pedigree of pigs. Therefore, the collection of diseased piglets is limited. Although the small number of individuals with the disease may limit the statistical power, our sample size is also representative in this study. In the future, the reliability of the results can also be improved by increasing the sample size.

## 5. Conclusions

This study aimed to explore candidate genes associated with intersex and aproctia in piglets. We conducted a GWAS analysis on 1030 Large White pigs and identified 15 genes in intersex and 4 genes in aproctia. Specifically, the *MAD1L1*, *ID4*, *EFNA5*, and *PPP1R16B* genes are, most likely, candidate genes for intersex, while the *ARNT2* gene is most likely a candidate gene for aproctia. These findings provide important clues for a deeper understanding of the genetic mechanisms of intersex and aproctia in pigs and provide new directions for the research and treatment of related congenital dysplasia. In breeding practice, the discovery of these key genes can be used in selective breeding to reduce the genetic transmission of adverse traits. Through genotyping or whole-genome selection, one can determine whether the breeding pigs carry risk alleles that cause intersex or anal lock, and marker-assisted selection can be used to eliminate or restrict their breeding use to avoid the accumulation of recessive mutations. In addition, while optimizing production performance and disease resistance, these risk genes can be included in the negative selection criteria to reduce the occurrence of intersex and aproctia and improve the growth performance and reproductive efficiency of the population. These research results not only provide a new direction for the genetic improvement of pig herds but also provide an important scientific basis for the research and treatment strategies of related congenital developmental abnormalities. In the future, by integrating genotyping, genomic selection, reproductive management, and epigenetics, breeding strategies can be further optimized, and combined with functional genomics and gene editing technology, more accurate genetic improvement tools can be provided for animal husbandry to promote healthy and efficient population cultivation.

## Figures and Tables

**Table 1:** Significant lead SNPs associated with intersex and aproctia in pigs identified by GWAS using 50 K chip and imputed data.

SSC ^a^	SNP ID	Position (bp) ^b^	Beta	*p*-Value	Nearest Gene	Data Types	
2	2_106861018_A	106861018	0.018	5.53 × 10^−10^	ST8SIA4	Imputed	Intersex
2	2_107619718_G	107619718	0.21	5.53 × 10^−10^	SLCO4C1		
2	2_112265453_C	112265453	0.21	5.53 × 10^−10^	EFNA5		
2	2_117364329_C	117364329	0.198	9.65 × 10^−9^	EPB41L4A		
3	3_1481804_A	1481804	0.183	1.81 × 10^−8^	MRM2, SNX8, EIF3B, ENSSSCG00000039023, CHST12, MAD1L1, NUDT1		
17	17_42520428_T	42520428	0.174	5.02 × 10^−8^	FAM83D, PPP1R16B, DHX35		
4	4_15237878_G	15237878	0.143	2.11 × 10^−6^	ID4	Chip	
7	7_49191557_A	49191557	0.116	1.84 × 10^−7^	ZFAND6, FAH, CTXND1, ARNT2	Imputed	Aproctia

^a^ Sus scrofa chromosome. ^b^ SNP position in Ensembl.

## Data Availability

The genotype and phenotype data are not publicly available yet due to planned future analyses but can be requested from the corresponding author.

## Supplementary Materials

The following supporting information can be downloaded at https://www.mdpi.com/article/10.3390/ani15081094/s1: Table S1: Imputed data GWAS identified SNPs significantly associated with pig intersex and their Fst results; Table S2: Chip data GWAS identified SNPs significantly associated with pig intersex and their Fst results; Table S3: Imputed data GWAS identified SNPs significantly associated with pig aproctia and their Fst results; Table S4: Chip data GWAS identified SNPs significantly associated with pig aproctia and their Fst results; Table S5: Pathway enrichment analysis reveals significantly enriched KEGG and GO pathways related to intersex; and Table S6: Pathway enrichment analysis reveals significantly enriched KEGG and GO pathways related to aproctia.

## References

[1] Eggers S., Sadedin S., Van Den Bergen J.A., Robevska G., Ohnesorg T., Hewitt J., Lambeth L., Bouty A., Knarston I.M., Tan T.Y. (2016). Disorders of sex development: Insights from targeted gene sequencing of a large international patient cohort. Genome Biol..

[2] Wu J., Yu H., Zhang Y., Zhao H., Zhong B., Yu C., Feng Z., Yu H., Li H. (2024). Pathological characteristics of SRY-negative 38, XX-DSD pigs: A family case report. Anim. Reprod. Sci..

[3] Hunter R., Greve T. (1996). Intersexuality in pigs: Clinical, physiological and practical considerations. Acta Vet. Scand..

[4] Pailhoux E., Pelliniemi L., Barbosa A., Parma P., Kuopio T., Cotinot C. (1997). Relevance of intersexuality to breeding and reproductive biotechnology programs; XX sex reversal in pigs. Theriogenology.

[5] Peretti V., Satué K., Ciotola F., Cristarella S., De Majo M., Biondi V., D’Anza E., Albarella S., Quartuccio M. (2020). An unusual case of testicular disorder in sex development of arabian mare (64, XX SRY-Negative). Animals.

[6] Szczerbal I., Komosa M., Nowacka-Woszuk J., Uzar T., Houszka M., Semrau J., Musial M., Barczykowski M., Lukomska A., Switonski M. (2021). A disorder of sex development in a Holstein–Friesian Heifer with a rare mosaicism (60, XX/90, XXY): A genetic, anatomical, and histological study. Animals.

[7] Knap P. (1986). Congenital defects inheritance of AI boars: Genetic parameters and breeding value estimation procedures. Livest. Prod. Sci..

[8] Thaller G., Dempfle L., Hoeschele I. (1996). Investigation of the inheritance of birth defects in swine by complex segregation analysis. J. Anim. Breed. Genet..

[9] Wiedemann S., Fries R., Thaller G. (2005). Genomewide scan for anal atresia in swine identifies linkage and association with a chromosome region on Sus scrofa chromosome 1. Genetics.

[10] Hori T., Giuffra E., Andersson L., Ohkawa H. (2001). Mapping loci causing susceptibility to anal atresia in pigs, using a resource pedigree. J. Pediatr. Surg..

[11] Jin Q., Wang C., Li X., Yu M., Zhao S.-H., Li X. (2013). Molecular characterization and genome-wide mutations in porcine anal atresia candidate gene GLI2. Mamm. Genome.

[12] Mulley R., Edwards M. (1984). Prevalence of congenital abnormalities in pigs. Aust. Vet. J..

[13] Norrish J., Rennie J. (1968). Observations on the inheritance of atresia ani in swine. J. Hered..

[14] Chang C.C., Chow C.C., Tellier L.C., Vattikuti S., Purcell S.M., Lee J.J. (2015). Second-generation PLINK: Rising to the challenge of larger and richer datasets. Gigascience.

[15] Zhou F., Wang S., Qin H., Zeng H., Ye J., Yang J., Cai G., Wu Z., Zhang Z. (2023). Genome-wide association analysis unveils candidate genes and loci associated with aplasia cutis congenita in pigs. BMC Genom..

[16] Ding R., Savegnago R., Liu J., Long N., Tan C., Cai G., Zhuang Z., Wu J., Yang M., Qiu Y. (2023). The SWine IMputation (SWIM) haplotype reference panel enables nucleotide resolution genetic mapping in pigs. Commun. Biol..

[17] Yang J., Lee S.H., Goddard M.E., Visscher P.M. (2011). GCTA: A tool for genome-wide complex trait analysis. Am. J. Hum. Genet..

[18] Yuan J., Kitchener A.C., Lackey L.B., Sun T., Jiangzuo Q., Tuohetahong Y., Zhao L., Yang P., Wang G., Huang C. (2024). The genome of the black-footed cat: Revealing a rich natural history and urgent conservation priorities for small felids. Proc. Natl. Acad. Sci. USA.

[19] Zhou X., Stephens M. (2012). Genome-wide efficient mixed-model analysis for association studies. Nat. Genet..

[20] Kanehisa M., Furumichi M., Sato Y., Kawashima M., Ishiguro-Watanabe M. (2023). KEGG for taxonomy-based analysis of pathways and genomes. Nucleic Acids Res..

[21] Bu D., Luo H., Huo P., Wang Z., Zhang S., He Z., Wu Y., Zhao L., Liu J., Guo J. (2021). KOBAS-i: Intelligent prioritization and exploratory visualization of biological functions for gene enrichment analysis. Nucleic Acids Res..

[22] Benjamini Y., Hochberg Y. (1995). Controlling the false discovery rate: A practical and powerful approach to multiple testing. J. R. Stat. Soc. Ser. B (Methodol.).

[23] Crew F. (1924). Hermaphroditism in the Pig. BJOG Int. J. Obstet. Gynaecol..

[24] Baker J.R. (1925). On sex-intergrade pigs: Their anatomy, genetics, and developmental physiology. J. Exp. Biol..

[25] Baker J.R. (1928). A new type of mammalian intersexuality. J. Exp. Biol..

[26] Brambell F.R. (1929). The histology of an hermaphrodite pig and its developmental significance. J. Anat..

[27] Johnston E., Zeller J., Cantwell G. (1958). Sex anomalies in swine. J. Hered..

[28] Breeuwsma A.J. (1971). Studies on intersexuality in pigs. Tijdschr. Voor Diergeneeskd..

[29] Basrur P., Kanagawa H. (1971). Sex anomalies in pigs. Reproduction.

[30] Booth W., Polge C. (1976). The occurrence of C19 steroids in testicular tissue and submaxillary glands of intersex pigs in relation to morphological characteristics. Reproduction.

[31] Hunter R., Baker T., Cook B. (1982). Morphology, histology and steroid hormones of the gonads in intersex pigs. Reproduction.

[32] Hunter R., Cook B., Baker T. (1985). Intersexuality in five pigs, with particular reference to oestrous cycles, the ovotestis, steroid hormone secretion and potential fertility. J. Endocrinol..

[33] Thomsen P., Poulsenm P. (1993). Analysis of the gonadal sex of five intersex pigs using Y chromosomal markers. Hereditas.

[34] Pailhoux E., Cotinot C., Popescu P., Boscher J., Legault C., Fellous M., Parma P., Molteni L. (1994). Genetic analysis of 38XX males with genital ambiguities and true hermaphrodites in pigs. Anim. Genet..

[35] Eyarefe O.D., Atawalna J., Emikpe B.O., Folitse R., Dei D., Duduyemi B., Okungbowa S., Okai D. (2017). Intersex piglet with bilobed urinary bladder in Kumasi, Ghana: A case report. Anim. Res. Int..

[36] Nowacka-Woszuk J., Szczerbal I., Stachowiak M., Szydlowski M., Nizanski W., Dzimira S., Maslak A., Payan-Carreira R., Wydooghe E., Nowak T. (2019). Association between polymorphisms in the SOX9 region and canine disorder of sex development (78, XX.; SRY-negative) revisited in a multibreed case-control study. PLoS ONE.

[37] Chan Y., Liu Y., Kong Y., Xu W., Zeng X., Li H., Guo Y., Tang X., Zhang J., Zhu B. (2023). Maternal genetic polymorphisms in the major mitotic checkpoint genes MAD1L1 and MAD2L1 associated with the risk of survival in abnormal chromosomal fetuses. Front. Genet..

[38] Bandala-Jacques A., Hernández-Cruz I.A., Castro-Hernández C., Díaz-Chávez J., Arriaga-Canon C., Barquet-Muñoz S.A., Prada-Ortega D.G., Cantú-de León D., Herrera L.A. (2020). Prognostic significance of the MAD1L1 1673 G: A polymorphism in ovarian adenocarcinomas. Rev. De Investig. Clin..

[39] Oatley J.M., Brinster R.L. (2008). Regulation of spermatogonial stem cell self-renewal in mammals. Annu. Rev. Cell Dev. Biol..

[40] Beger C., Pierce L.N., Krüger M., Marcusson E.G., Robbins J.M., Welcsh P., Welch P.J., Welte K., King M.-C., Barber J.R. (2001). Identification of Id4 as a regulator of BRCA1 expression by using a ribozyme-library-based inverse genomics approach. Proc. Natl. Acad. Sci. USA.

[41] Dong J., Huang S., Caikovski M., Ji S., McGrath A., Custorio M.G., Creighton C.J., Maliakkal P., Bogoslovskaia E., Du Z. (2011). ID4 regulates mammary gland development by suppressing p38MAPK activity. Development.

